# NDUFV1 attenuates renal ischemia–reperfusion injury by improving mitochondrial homeostasis

**DOI:** 10.1111/jcmm.17735

**Published:** 2023-04-07

**Authors:** Lu Li, Lingling Zhang, Yingjie Cao, Xu Chen, Haifeng Gong, Yidan Ma, Yuanyuan Gui, Tianya Xiang, Jianxing Liu, Xinzhong Huang

**Affiliations:** ^1^ Department of Nephrology Affiliated Hospital of Nantong University 20 Xisi Road Nantong Jiangsu 226001 China; ^2^ Medical School of Nantong University Nantong 226001 China

**Keywords:** acute kidney injury, cell apoptosis, mitochondrial homeostasis, NDUFV1, oxidative stress

## Abstract

Impaired mitochondrial function and dysregulated energy metabolism have been shown to be involved in the pathological progression of kidney diseases such as acute kidney injury (AKI) and diabetic nephropathy. Hence, improving mitochondrial function is a promising strategy for treating renal dysfunction. NADH: ubiquinone oxidoreductase core subunit V1 (NDUFV1) is an important subunit of mitochondrial complex I. In the present study, we found that NDUFV1 was reduced in kidneys of renal ischemia/reperfusion (I/R) mice. Meanwhile, renal I/R induced kidney dysfunction as evidenced by increases in BUN and serum creatinine, severe injury of proximal renal tubules, oxidative stress, and cell apoptosis. All these detrimental outcomes were attenuated by increased expression of NDUFV1 in kidneys. Moreover, knockdown of *Ndufv1* aggravated cell insults induced by H_2_O_2_ in TCMK‐1 cells, which further confirmed the renoprotective roles of NDUFV1. Mechanistically, NDUFV1 improved the integrity and function of mitochondria, leading to reduced oxidative stress and cell apoptosis. Overall, our data indicate that NDUFV1 has an ability to maintain mitochondrial homeostasis in AKI, suggesting therapies by targeting mitochondria are useful approaches for dealing with mitochondrial dysfunction associated renal diseases such as AKI.

## 1 INTRODUCTION

Acute kidney injury (AKI) is a disease with high mortality and morbidity.^1^ The causes of AKI are various, among them, septicemia, low perfusion, and nephrotoxic injury are the most common risk factors.^2^ Low perfusion injury is a hallmark of AKI, which is also a main leading cause for chronic kidney disease (CKD).^3^, ^4^ The severity, duration, frequency, and age determine whether AKI develops into CKD.^5^ Many events have been shown to be involved in the pathogenesis of AKI, such as inflammatory response, renal hypoperfusion, oxidative stress, mitochondrial dysfunction, and microcirculation disturbance. However, there is still a lack of effective targets for treating AKI and CKD.^6^, ^7^

Due to large amount substance exchange, the kidney is one of the most energy‐demanding organs in the body.^8^ One possible mechanism of AKI damage is the destruction of mitochondrial homeostasis.^9^ It has been shown that mitochondrial dysfunction is involved in the pathogenesis of AKI and triggers the progression from AKI towards CKD.^10^, ^11^, ^12^, ^13^ Proximal renal tubular dysfunction caused by AKI reduced the activity of mitochondrial complex I and the production of adenosine triphosphate (ATP).^14^ In accordance, restoring the function and homeostasis of mitochondria is a feasible strategy to prevent AKI.^15^ Therefore, the control of mitochondrial dynamics is likely a useful strategy for dealing with kidney dysfunction induced by ischemia and hypoxia.^16^ Complex I (NADH: ubiquinone oxidoreductase) is the first enzyme complex in the mitochondrial respiratory chain, which consists of 45 subunits.^17^ Its dysfunction impairs the mitochondrial function in renal tubules during reperfusion, which is often associated with energy deficiency.^18^ NDUFV1 is the nuclear‐encoded structural subunit of complex I. NDUFV1 mutations have been shown to be associated with Leigh syndrome (LS), Leigh‐like syndrome (LL), diffuse leukoencephalopathy, and Parkinson's disease.^19^ We therefore proposed that NDUFV1 may hold therapeutic potential for treating AKI in ischemia–reperfusion (I/R) model mice.

In the present study, we increased NDUFV1 expression in the kidney to examine the renoprotective roles of NDUFV1 in renal I/R mice. Our results showed that the reinforcement expression of NDUFV1 has multiple benefits against kidney damages induced by renal I/R. For example, NDUFV1 reduces serum creatinine and blood urea nitrogen (BUN), attenuates proximal tubule injury, and mitigates cell apoptosis. All these benefits are likely due to the increased integrity and function of mitochondria, and reduced oxidative stress by NDUFV1. These data indicate that NDUFV1 plays an essential role for the proper functioning of the kidney, and for this reason, NDUFV1 might be a target for treating kidney diseases such as AKI and CKD.

## 2 MATERIALS AND METHODS

### 2.1 Animal model

And 8‐week‐old male C57BL/6 mice were provided by the Animal Center of Nantong University. Mice were randomly divided into three groups: sham, renal ischemia/reperfusion (I/R) and I/R + NDUFV1 group. The plasmid expressing *Ndufv1* was provided by Dr. Cheng Sun.^20^ To increase NDUFV1 expression in the kidney, mice received 10 μg of the plasmid expressing *Ndufv1* via tail vein injection in 8–12 s.^21^ Mice in control group received 10 μg of empty vector in 8–12 s. And 48 h post‐plasmid injection, mice were subjected to renal I/R surgery using bilateral renal pedicle ligation as described elsewhere with slight changes.^22^ Briefly, after anaesthetization using pentobarbital sodium, renal artery and vein were exposed and blood flow was occluded using a microvascular clamp for 30 min. Mice in sham group were subjected to similar operations without blood vessel clamping. Kidney and blood samples were collected at 12 h, 24 h, or 36 h post‐surgery for further processing. No exclusion criteria were pre‐determined to exclude animals. Sample sizes were based on our pilot experiments, in which 5 mice in each group provided sufficient statistical power. No blinding was done in animal experiments. All the procedures were approved by the Animal Care and Use Committee of Nantong University and the Jiangsu Province Animal Care Ethics Committee (Approval ID: SYXK [SU] 2017‐0046).

### 2.2 TCMK‐1 cell culture and *Ndufv1* knockdown

TCMK‐1 cells were obtained from the American Type Culture Collection (ATCC; CCL‐139) and cultured in DMEM/F12 medium containing 10% fetal bovine serum at 37°C in 5% CO_2_ atmosphere. Cells were authenticated by STR profiling and tested for mycoplasma contamination. To knockdown *Ndufv1* expression, cells were transfected with siRNAs targeting *Ndufv1* by riboFECT™CP (RiboBio). *Ndufv1* siRNAs were designed and synthesized at RiboBio. The sequences for each siRNA are, GGT CTG ATC CGA CAT TTC A (siRNA‐1); CGC GCT GCC TAT ATC TAC A (siRNA‐2); CAA GGA CCG GGA GAT CTT A (siRNA‐3). Cells in control group were transfected with negative control (NC) (RiboBio), which does not target any known mammalian genes. To induce oxidative stress, cells were treated with 500 μM of H_2_O_2_ for 24 h.

### 2.3 Detection of intracellular reactive oxygen species

And 2′,7′‐dichlorofluorescin diacetate (DCFH‐DA; Beyotime) was used to detect intracellular reactive oxygen species (ROS). Briefly, DCFH‐DA was dissolved in serum‐free medium at a concentration of 10 μM and used as working solution. To detect ROS, cells were incubated with DCFH‐DA working solution for 20 min at 37°C in dark. After three‐time washing in serum‐free medium, fluorescence in cells was monitored and captured with a fluorescence microscope. Fluorescence intensity was quantified by using the ImageJ software.

### 2.4 Assays of ATP, glutathione, malondialdehyde, and protein carbonyl

ATP content was analysed by an ATP Bioluminescence Assay Kit from Jiancheng Bioengineering Institute (A095‐2‐1). Glutathione (GSH), malondialdehyde (MDA), and protein carbonyl (PC) were measured by commercial kits from Jiancheng Bioengineering Institute (A006‐2‐1; A003‐4‐1; A087‐1‐2) according to the manufacturer's instructions.

### 2.5 Measurements of GSH‐PX and SOD activities

Glutathione peroxidase and SOD activities were analysed by using commercial kits form Jiancheng Bioengineering Institute (A005‐1‐2; A001‐3‐2) according to the manufacturer's instructions.

### 2.6 Measurement of complex I activity

Complex I activity was analysed by using a commercial kit (Abcam; ab109721) according to the manufacturer's instructions.

### 2.7 Measurements of serum creatinine and BUN

A creatinine assay kit and a BUN assay kit (Shanghai Kehua Bioengineering Co., Ltd.) were used for measuring serum creatinine and BUN, respectively. All detections were conducted according to the instructions from the manufacturer.

### 2.8 Kidney histological examination

Kidney tissues were taken out and immediately fixed in 4% paraformaldehyde/phosphate buffer saline (PBS) and then were embedded in paraffin. The embedded paraffins were cut into 4‐μm thin slices for Periodic Acid‐Schiff (PAS) staining, and the structural changes of the kidney were observed under a light microscope. For renal tubular injury index, the renal tubules in a visual field on PAS staining sections with ×400 magnification were used for semi‐quantitative evaluation from 0 to 4 (0, normal interstitium; 1: injured area <25%; 2: injured area from 26% to 50%; 3: injured area from 51% to 75%; 4: injured area >75%).^23^

### 2.9 RNA extraction and quantitative real‐time PCR (qRT‐PCR)

Total RNA was extracted from renal tissues using TRIzol reagent (Invitrogen). Reverse transcription was performed using a first‐strand cDNA Reverse Transcription Kit (Takara). Real‐time polymerase chain reaction was performed using a SYBR Premix Ex Taq II (Tli RNase HPlus) Kit according to the manufacturer's instructions (RR820; Takara). The primers used were as follows: 18 s ribosomal RNA (rRNA): 5′‐AGT CCC TGC CCT TTG TTT GTA‐3′ (forward) and 5′‐CGT TCC AGG GCC TCA C‐3′ (reverse). *mt‐Nd1*: 5′‐AAT CGC CAT AGC CTT GCT AAC AT‐3′ and 5′‐GGC GTC TGC AAA TGG TTG TAA‐3′ (reverse). *Ndufv1*: 5′‐TTT CTC GGC GGG TTG GTT C‐3′ (forward) and 5′‐GGT TGG TAA AGA TCC GGT CTT C‐3′ (reverse). The expression for the messenger RNA (mRNA) of interest was calculated with a comparative method (2^−∆∆Ct^) normalized to 18 s rRNA.

### 2.10 Western blot analysis

Kidney tissues were subjected to western blot analysis using a procedure described previously.^24^ After blocking nonspecific binding with 5% nonfat milk in phosphate‐buffered saline solution or 1 h at room temperature, membranes were incubated with the primary antibodies overnight at 4°C. The primary antibodies include anti‐NDUFV1 antibody (PA5‐98007; Invitrogen), anti‐SDHA antibody (11,998; Cell Signalling Technology), anti‐HSP60 antibody (12,165; Cell Signalling Technology), anti‐PHB1 antibody (2426; Cell Signalling Technology), anti‐VDHC antibody (4661; Cell Signalling Technology), anti‐Cox IV antibody (4850; Cell Signalling Technology), anti‐Bax antibody (2772; Cell Signalling Technology), anti‐Bcl‐2 antibody (3498; Cell Signalling Technology), anti‐cleaved Caspase 3 antibody (9664; Cell Signalling Technology), and anti‐Actin antibody (3700; Cell Signalling Technology). After three times washing in Tris‐buffered saline with Tween (TBST), membranes were then incubated with an appropriate secondary antibody for 1 h at room temperature. Membranes were developed using a chemiluminescence reagent. The Image J software was used to analyse the densitometry of the blots.

### 2.11 Immunostaining

Immunostaining was performed using a procedure described previously.^25^ Paraffin‐embedded kidney sections (4 μm) were deparaffinized, and ethylene diamine tetra acetic acid (1 mM) was used for antigen retrieval. The slides were incubated with anti‐Caspase 3 antibody (1:200; ab238440, Abcam) at 4°C overnight. After 3‐time washing in PBS, the slides incubated with fluorescent secondary antibody for 1 h at room temperature. After removing the second antibody, the slides were immersed in solution containing DAPI (D9542; Sigma‐Aldrich) for 5 min to label the nuclei. The images were detected by fluorescence microscopy, and positive images were analysed by computerized digital image analysis. The intensity of immunofluorescence was analysed using the software of ImageJ.

### 2.12 TUNEL staining

Apoptosis in renal tissues was identified by a TUNEL assay with an in‐situ Cell Death Detection Kit (11684817910; Roche). The assay was performed according to the manufacturer's instructions.

### 2.13 Succinic dehydrogenase activity staining

The procedures for succinic dehydrogenase (SDH) activity staining were described elsewhere.^26^ Briefly, kidney tissues were cut into 10 μm thick cross sections by using a cryostat‐microtome (Leica, CM3050S). The sections were then incubated with SDH working solution containing 20 mM KH_2_PO_4_, 76 mM Na_2_HPO_4_, 5.4% succinic acid disodium salt, 0.02% NBT for 1 h at 37°C. The sections were rinsed three times in PBS and fixed in 10% formalin for 10 min at room temperature. After three time washing with 15% ethanol, images for SDH staining were taken with a microscope.

### 2.14 Mitochondrial ultra‐structure analysis

To observe mitochondrial ultra‐structures, a transmission electron microscope was employed to this aim using a previous procedure.^26^ Briefly, kidney samples were pre‐fixed in cacodylate buffer (0.1 M, pH 7.4), in which paraformaldehyde (2.5%) and glutaraldehyde (2.5%) were included. After that, samples were transferred into osmium tetraoxide (1%) and incubated for 1 h at 4°C. After gradient dehydration in alcohol, samples were oriented longitudinally and embedded in Epon 812, and cut into 70 nm thickness sections, which were then contrasted in lead citrate and uranyl acetate. Finally, these prepared sections were analysed with a transmission electron microscope at 80 kV (JEO Ltd.).

### 2.15 Statistical analysis

No animals or samples were excluded from the analysis. The data were expressed as mean ± SEM with the results of three independent experiments. One‐way analysis of variance (anova) followed by Bonferroni's post hoc test was used for comparison in more than two groups. *p* < 0.05 was considered statistically significant.

## 3 RESULTS

### 3.1 NDUFV1 attenuates renal injury induced by I/R in mice

**FIGURE 1:**
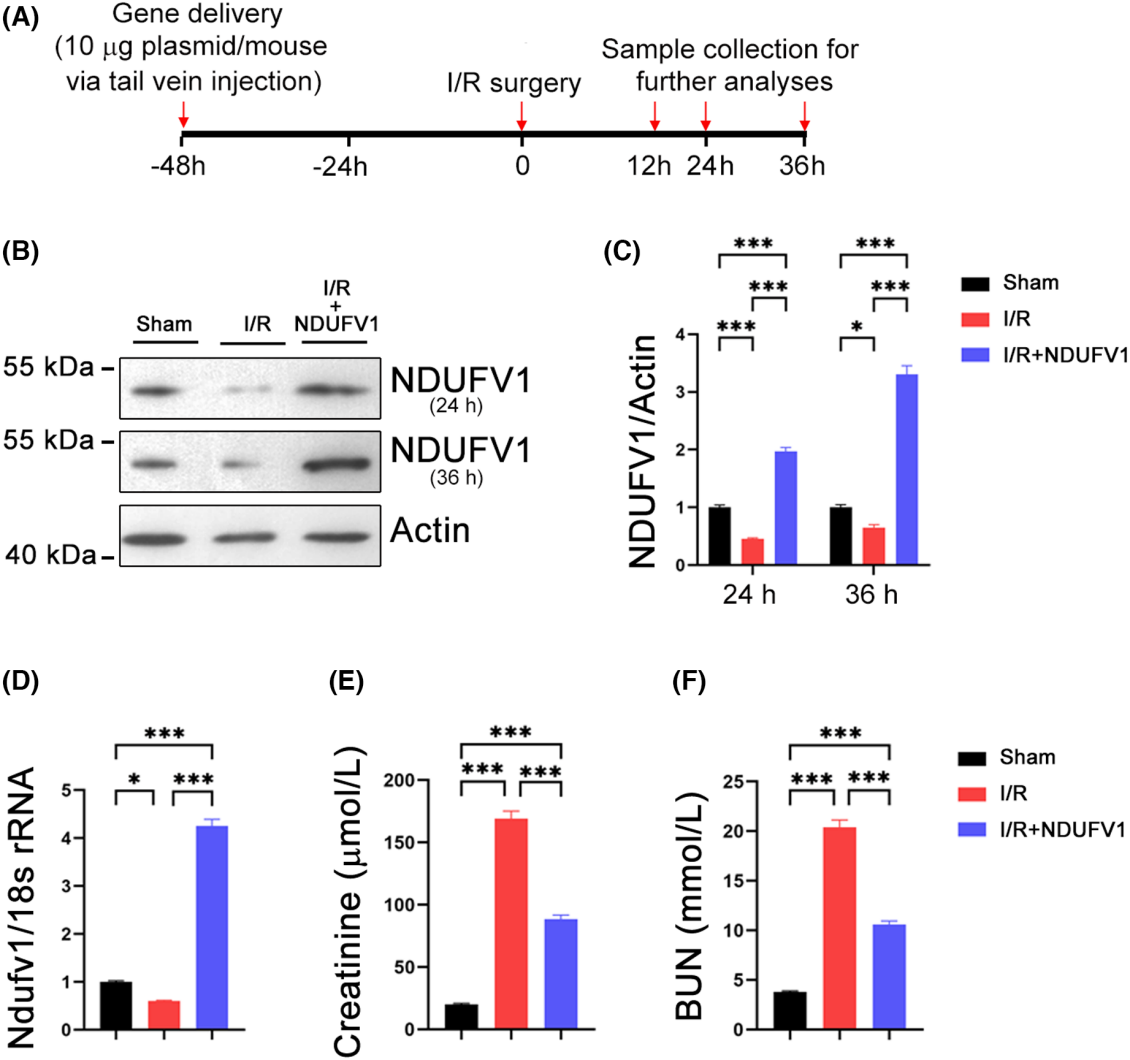
NDUFV1 attenuates kidney dysfunction in renal ischemia/reperfusion (I/R) mice. To increase NDUFV1 expression in the kidney, 10 μg of plasmid expressing *Ndufv1* was delivered to mice via tail vein injection. 48 h post‐injection, mice were subjected to I/R surgery to induce renal failure. Samples were collected at different time points for further analyses. (A) The diagram for in vivo experiments. (B) The protein levels of NDUFV1 was reduced by I/R in the kidney. Kidneys were collected at 24 h or 36 h post‐surgery for protein assay using western blot and Actin was used as a loading control. (C) Quantification of western blot data as shown in (B). (D) The mRNA levels of NDUFV1 was decreased in I/R mouse kidneys. Kidneys were collected at 36 h post‐surgery for gene expression analysis using qRT‐PCR and 18 s rRNA was used as an internal control. (E, F) Serum creatinine and BUN levels. Blood samples were collected at 36 h post‐surgery, serum creatinine levels (E) and BUN (F) were assayed. Data are shown as means ± SEM (*n* = 5). *p < 0.05, ***p < 0.001, by one‐way anova.

**FIGURE 2:**
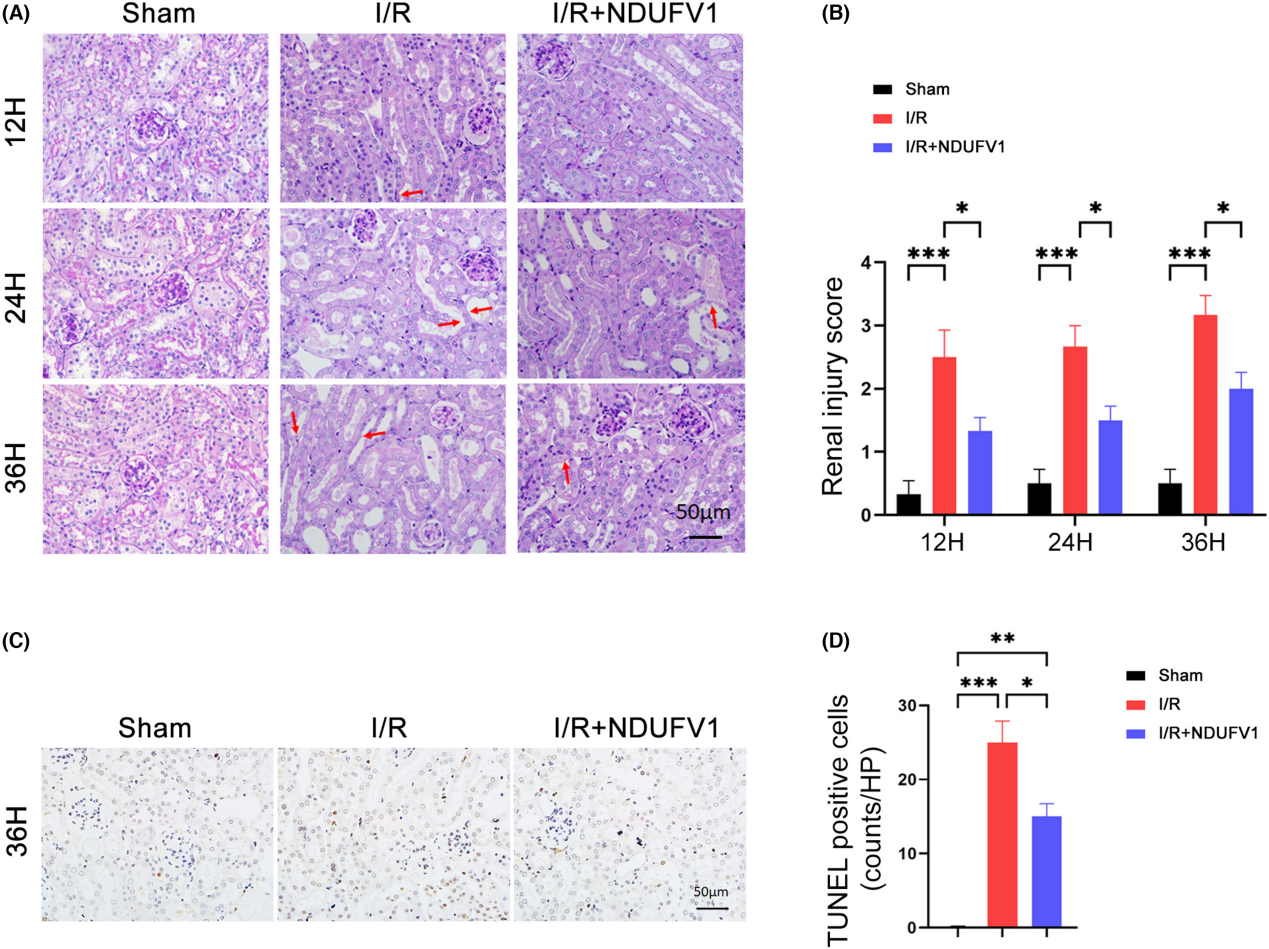
NDUFV1 attenuates renal injury induced by renal I/R. (A) Periodic Acid‐Schiff (PAS) staining. Arrowheads indicate dilated tubules and brush‐border damage after renal I/R. Kidneys were collected at 12 h, 24 h, or 36 h post‐surgery and subjected to PAS staining. (B) Semi‐quantitative analysis of PAS staining as shown in (A). (C) TUNEL staining. (D) Quantification of TUNEL positive cells. Data are shown as means ± SEM (*n* = 5). *p < 0.05, **p < 0.05, ***p < 0.001, by one‐way anova.

To test the potential role of NDUFV1 in the kidney, we increased NDUFV1 expression in mouse kidneys by tail vein injection with a plasmid expressing *Ndufv1*, and then examined renal function (Figure 1A). First, we analysed the expression of NDUFV1. As shown in Figure 1B,C, the protein levels of NDUFV1 in kidneys were markedly decreased at 24 h and 36 h post‐I/R surgery; the application of plasmid expressing *Ndufv1* successfully restored this decline, its expression was even higher than that in sham group (Figure 1B,C). Furthermore, similar changes were observed in the mRNA levels of NDUFV1 (Figure 1D). These findings suggest that reduced expression of NDUFV1 in kidneys might be involved in renal injury induced by I/R, whereas reinforcement of NDUFV1 may play a benefit role against renal I/R‐induced damages. In addition, the current strategy using a hydrodynamic‐based method can increase exogenous NDUFV1 expression in mouse kidneys. Of note, it has been shown that exogenous gene was predominantly expressed in renal glomeruli and tubules using this gene delivery method.^27^ Next, we examined whether NDUFV1 plays a beneficial role in AKI. To this end, blood samples were collected at 36 h post‐I/R surgery. In I/R model mice, the renal failure was characterized by significant increases in creatinine and BUN (Figure 1E,F). However, in the presence of exogenous NDUFV1, these detrimental changes were largely prevented (Figure 1E,F). Moreover, we examined the pathological changes of kidney proximal renal tubules by PAS staining. The results showed that the epithelial cells of renal tubules in I/R model mice were flattened, the basement membrane was exposed, and the lumen was dilated (Figure 2A,B). These damages were alleviated by overexpression of NDUFV1 in kidneys (Figure 2A,B). In summary, increased expression of NDUFV1 in the kidney is a useful strategy for improving renal function in AKI.

### 3.2 NDUFV1 represses renal apoptosis in I/R model mice

**FIGURE 3:**
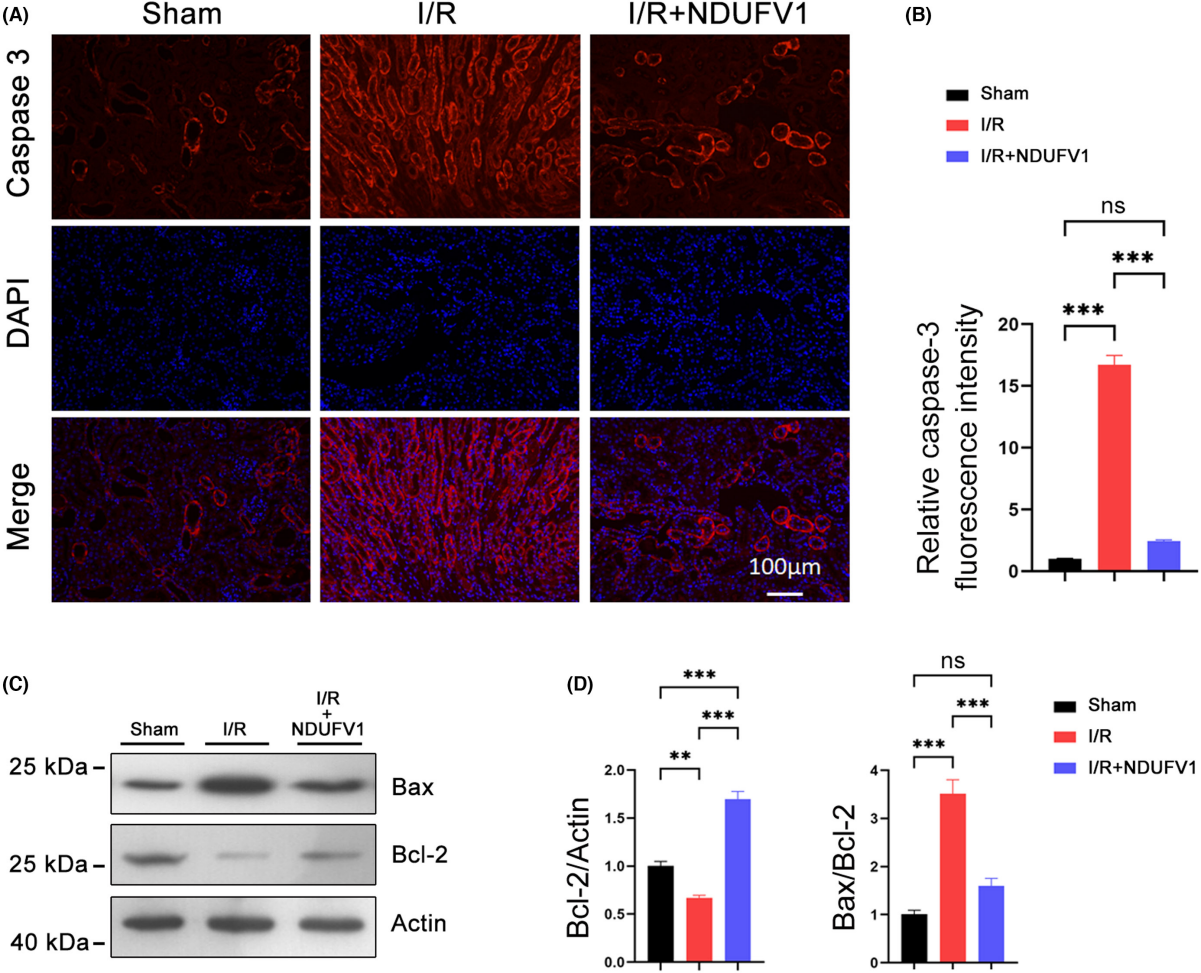
NDUFV1 represses renal tubular epithelial cell apoptosis induced by renal I/R. (A) Immunostaining for analysing cleaved Caspase‐3 expression in kidneys. Scale bar = 100 μm. (B) Quantification of fluorescence intensity for cleaved Caspase‐3 as shown in (A). (C) Western blot analysis showing reduced Bax and enhanced Bcl‐2 by NDUFV1 in kidneys. Actin was used as a loading control. (D) Relative protein levels of Bcl‐2 and Bax/Bcl‐2. Data are shown as means ± SEM (*n* = 5). ns: no significance, **p < 0.05, ***p < 0.001, by one‐way anova.

It has been shown that renal cell apoptosis is often associated with the pathological progression of AKI.^28^ In accordance, we observed renal tubule epithelial cell apoptosis was increased by renal I/R as evidenced by the increased TUNEL positive cells (Figure 2C,D). In the presence of exogenous *Ndufv1*, the cell apoptosis induced by renal I/R was largely prevented (Figure 2C,D). Furthermore, the expression of cleaved Caspase‐3 was dramatically induced by renal I/R; overexpression of NDUFV1 attenuated this trend (Figure 3A,B). Meanwhile, we analysed the expression of Bax and Bcl‐2, two cell apoptosis‐related proteins. As shown in Figure 3C, Bax was increased by renal I/R, and this increase was largely mitigated by NDUFV1. On the contrary, Bcl‐2 was decreased by renal I/R, NDUFV1 prevented this decline (Figure 3C,D). As a result, the value for Bax/Bcl‐2 was significantly increased in I/R group, which was markedly blunted by NDUFV1 (Figure 3D). These data clearly indicate that NDUFV1 can reduce renal cell apoptosis induced by renal I/R in mice.

### 3.3 NDUFV1 improves the integrity of mitochondria in renal I/R model mice

**FIGURE 4:**
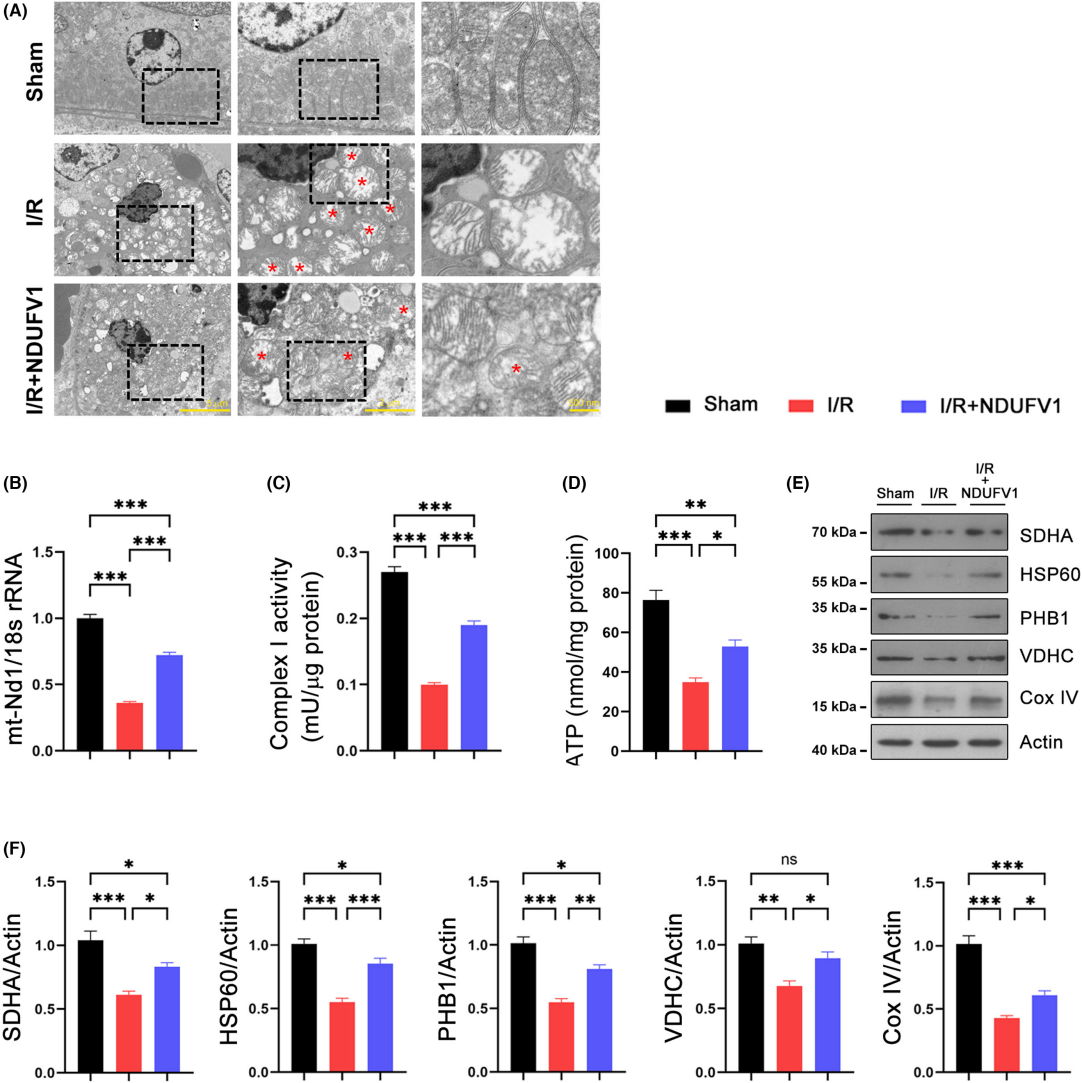
NDUFV1 improves the integrity of mitochondria in kidneys of renal I/R mice. (A) The ultra‐structures of mitochondria were analysed by transmission electron microscopy. The squared areas were magnified in right panel. Scale bar = 5 μm in the left panel; Scale bar = 2 μm in the middle panel; Scale bar = 500 nm in the right panel. Red star was used to indicate damaged mitochondria. (B) Mitochondrial DNA copy number. (C) The mitochondrial complex I activity. (D) ATP contents. (E) The protein levels of succinate dehydrogenase (SDHA), heat shock protein 60 (HSP60), prohibitin 1 (PHB1), voltage‐dependent anion channel (VDHC), and cytochrome c oxidase IV (Cox IV). Protein levels were analysed by western blot and Actin was used as a loading control. (F) Quantification of western blot data as shown in (E). Data are shown as means ± SEM (*n* = 5). ns: no significance, *p < 0.05, **p < 0.01, ***p < 0.001, by one‐way anova.

**FIGURE 5:**
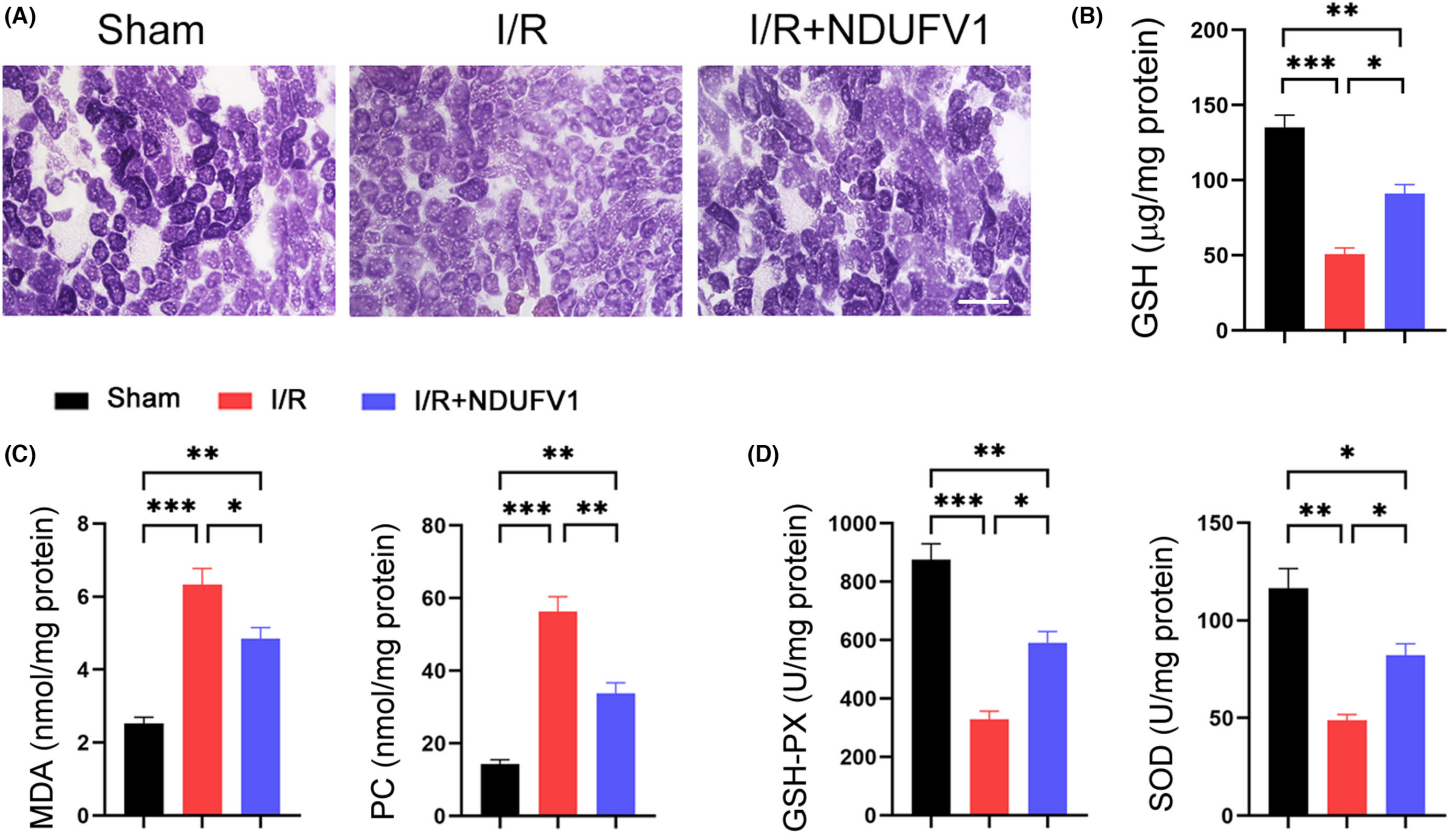
NDUFV1 ameliorates oxidative stress induced by renal I/R. (A) Succinate dehydrogenase (SDH) staining. Scale bar = 100 μm. (B) GSH assay. (C) Malondialdehyde (MDA) and protein carbonyl (PC) assays. (D) The activities of glutathione peroxidase (GSH‐PX) and superoxide dismutase (SOD). Data are shown as means ± SEM (*n* = 5). *p < 0.05, **p < 0.05, ***p < 0.001, by one‐way anova.

Mitochondrial dysfunction is a common event for renal impairment induced by I/R.^13^ We therefore examined mitochondrial function in the following experiments. The observed ultra‐structures using a transmission electron microscope (TEM) revealed that many mitochondria in kidneys were damaged by I/R surgery, as evidenced by swelled volume, disrupted crista, and smeared double membrane structure; and these detrimental outcomes were largely attenuated by NDUFV1 (Figure 4A). The mitochondrial DNA copy number was decreased by renal I/R; overexpression of NDUFV1 prevented this decline (Figure 4B). The activity of complex I was reduced in kidneys of I/R mice. The forced expression of NDUFV1 partially restored complex I activity (Figure 4C). The production of ATP in kidneys was reduced by renal I/R; NDUFV1 attenuated this decline (Figure 4D). Furthermore, we analysed the protein levels of SDHA, HSP60, PHB1, VDHC, and Cox IV, which belong to other electron transport chain complexes. The results showed that all these proteins were decreased by renal I/R; whereas overexpression of NDUFV1 exhibited benefits against these declines (Figure 4E,F). Succinate dehydrogenase (SDH) staining showed that NDUFV1 ameliorated the decrease in SDH activity in kidneys (Figure 5A). These data indicate that increased expression of NDUFV1 improves the integrity of the mitochondrial electron transport chain complexes in renal I/R mice.

### 3.4 NDUFV1 mitigates oxidative stress in kidneys induced by renal I/R

Mitochondria have been revealed as a main organelle for producing ROS in cells, which may eventually cause oxidative stress.^29^ Therefore, we next examined oxidative stress in kidneys. As shown in Figure 5B, the content of GSH was decreased by renal I/R, whereas NDUFV1 prevented this decline. The increased levels in malondialdehyde (MDA) and protein carbonyl (PC) induced by renal I/R were markedly repressed by NDUFV1 (Figure 5C). The activities of glutathione peroxidase (GSH‐PX) and superoxide dismutase (SOD) were reduced in I/R mouse kidneys. Reinforced expression of NDUFV1 partially rescued these enzyme activities (Figure 5D).

### 3.5 Knockdown of *Ndufv1* aggravates oxidative stress induced by H_2_O_2_ in TCMK‐1 cells

**FIGURE 6:**
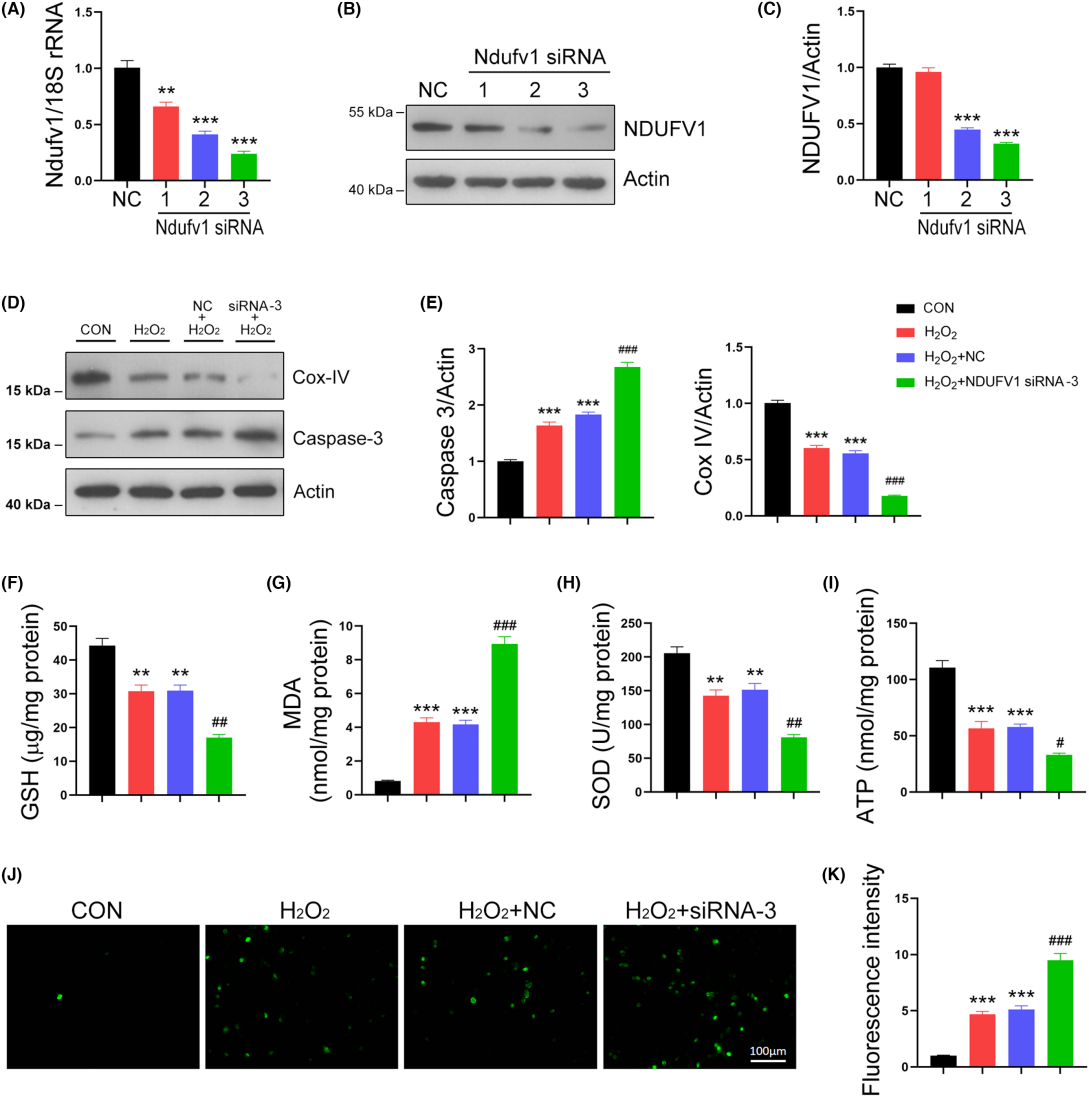
Knockdown of *Ndufv1* aggravates oxidative stress in TCMK‐1 cells. (A, B) Knockdown of *Ndufv1* by siRNAs. Cells were transfected with *Ndufv1* siRNAs for 72 h. The expression of *Ndufv1* was analysed by qRT‐PCR (A) and western blot (B). 18S rRNA was used as an internal control in qRT‐PCR, and Actin was used as a loading control in western blot. (C) Quantification of western blot data as shown in (B). (D) Effects of *Ndufv1* knockdown on the expressions of Cox‐IV and cleaved Caspase 3. Cells were transfected with *Ndufv1* siRNA‐3 for 48 h, and then cells were treated with 500 μM of H_2_O_2_ for 24 h. Total cell lysates were prepared and subjected to western blot analysis. Actin was used as a loading control. (E) Quantification of Cox‐IV and cleaved Caspase 3 as shown in (D). (F–I) ATP (F), GSH (G), MDA (H), and SOD activity (I) assays. (J) The intracellular production of ROS. (K) Relative fluorescence intensity of ROS as shown in (J). CON, control; NC, negative control; ROS, reactive oxygen species. Data are shown as means ± SEM (*n* = 5). **p < 0.01, ***p < 0.001 versus NC or CON; ^#^ *p* < 0.05, ^##^ *p* < 0.01, ^###^ *p* < 0.001 versus H_2_O_2_ + NC, by one‐way anova.

The above gain‐of‐function experiments showed the benefits of NDUFV1 on renal function, next we further examined the renoprotective role of NDUFV1 by loss‐of‐function studies. To this end, TCMK‐1 cells were transfected with siRNAs against *Ndufv1* to reduce *Ndufv1* expression. Indeed, the mRNA levels of *Ndufv1* were decreased by all three tested siRNAs (Figure 6A). Western blot data further confirmed the knockdown efficiencies of *Ndufv1* siRNAs (Figure 6B,C). Of these siRNAs, siRNA‐3 exhibited the best knockdown efficiency and it was chosen for the following experiments. As shown in Figure 6D, the expression of Cox‐IV was decreased by H_2_O_2_; *Ndufv1* knockdown further aggravated this trend. Meanwhile, cleaved Caspase‐3 was increased by H_2_O_2_, which was further fortified in *Ndufv1* siRNA‐3 transfected cells (Figure 6D,E). The levels of GSH were reduced by H_2_O_2_; knockdown of *Ndufv1* further reduced GSH (Figure 6F). The increase in MDA induced by H_2_O_2_ was strengthened by *Ndufv1* siRNA‐3 (Figure 6G). SOD activity was declined in the presence of H_2_O_2_, particularly in *Ndufv1* knockdown cells (Figure 6H). The reduced production of ATP by H_2_O_2_ was further declined by *Ndufv1* siRNA‐3 (Figure 6I). Moreover, the intracellular production of ROS was increased by H_2_O_2_; knockdown of *Ndufv1* further enhanced this induction (Figure 6J,K). These data further confirmed the essential role of NDUFV1 in mitochondrial function under oxidative stress.

## 4 DISCUSSION

In the present study, we found that increased expression of NDUFV1 in the kidney plays a renoprotective role in I/R model mice. For example, NDUFV1 reduces the increased BUN and serum creatinine, attenuates proximal renal tubule injury, and ameliorates cell apoptosis in kidneys. As a member of mitochondrial complex I, NDUFV1 improves mitochondrial function and homeostasis, thus plays a beneficial role against oxidative stress and defected energy supply in renal I/R. These data strongly suggest that NDUFV1, together with other members of complex I, may hold therapeutic potential for treating kidney diseases such as AKI.

In the human body, the kidney is a secondly energy‐demanding organ, due to it requires larger amount energy to remove waste from the blood, reabsorb nutrients, regulate the balance of electrolytes and fluid, maintain acid–base balance, and manipulate blood pressure. Therefore, mitochondrial homeostasis is extremely important for the proper functioning of the kidney. It is reasonable to predict that, therefore, maintaining mitochondrial function in the kidney is a promising strategy for treating renal failure. Complex I in mitochondria is the entry point for electrons from NADH into the respiratory chain. It is the largest and most complicated complex of the respiratory chain in mitochondria. Except its central role in the construction of proton‐motive force, it is a major contributor to cellular production of ROS. At this regard, disruption of complex I is often associated with a variety of detrimental outcomes. For example, complex I defects induced by *Ndufv1* mutations have been shown to be involved in several disorders including encephalopathy, myopathy, and Leigh syndrome.^30^, ^31^, ^32^ Moreover, NDUFS1 (NADH:ubiquinone oxidoreductase 75 kDa Fe‐S protein 1) is another member of complex I, its mutations also correlated with various diseases such as diabetic cardiomyopathy, myocardial hypertrophy, schizophrenia, leukoencephalopathy, and Leigh syndrome.^33^, ^34^, ^35^, ^36^, ^37^ Most recently, deficiency of *Ndufs2* in dopaminergic neurons caused Parkinson's disease‐like phenotypes in mice.^38^ It is worthy to note that, all these diseases mentioned above happen in the brain or heart, both organs are energy‐demanding tissues. In the present study, we found that *Ndufv1* expression was markedly reduced in kidneys from renal I/R mice. Moreover, knockdown of *Ndufv1* in TCMK1‐cells further aggravated oxidative stress induced by H_2_O_2_. These evidences implied that complex I is involved in the progression of AKI. In line with this prediction, deficiency in complex I induced by interruption of *Ndufs6* induced renal injury including albuminuria and renal fibrosis.^39^ Therefore, these evidences strongly suggest that complex I is a promising target for dealing with mitochondrial defects associated disorders including kidney diseases.

In line with the notion mentioned above, reinforcement of complex I seems to be a useful approach for treating mitochondrial dysfunction‐related diseases. Indeed, forced expression of *Ndufs1* in cardiocytes improves complex I activity and alleviates cardiac dysfunction and myocardial fibrosis.^40^ HGC is a newly designed HDAC6 inhibitor, which targets NDUFV1 to attenuate cell injury in dopaminergic neurons treated with neurotoxin MPP^+^.^20^ Furthermore, the recovery of mitochondrial function can accelerate the recovery of podocytes after glomerular injury.^41^ In the present study, accordingly, we observed that overexpression of NDUFV1 in the kidney plays a beneficial role in renal I/R mice, as evidenced by decreased BUN and serum creatinine, alleviated proximal renal tubule injury, and reduced cell apoptosis. Our data further confirmed that complex I is a promising therapeutic target for developing drugs against kidney diseases such as AKI. Of note, defects in complex III and IV also have been found to be involved in the pathogenesis of renal dysfunction.^42^, ^43^ Therefore, these complexes, as well as complex I, might be therapeutic targets for treating renal failure induced by energy deficiency.

In addition to energy supply, mitochondria also produce ROS as byproducts. Excessive ROS accumulation in the cell may cause detrimental outcomes such as cytochrome c release and caspase activation, and eventually induces cell apoptosis. Indeed, we found that renal I/R induced cleaved caspase‐3 expression and cell apoptosis in kidneys. These detrimental outcomes could be ameliorated by NDUFV1, suggesting NDUFV1 improves the integrity and function of mitochondria. The TEM and biochemical analyses revealed that the mitochondrial integrity and function in kidneys were improved by NDUFV1.

Furthermore, we observed that increased expression of NDUFV1 plays a beneficial role in the ROS defence system, as evidenced by the increased activities of SOD and GSH‐PX in NDUFV1‐overexpressed kidneys. As we know, SOD converts superoxide anions to hydrogen peroxide (H_2_O_2_) and oxygen, whereas GSH‐PX reduces H_2_O_2_ to H_2_O. Both SOD and GSH‐PX have shown renoprotective effects in AKI.^44^ Therefore, activation of SOD and GSH‐PX is likely an alternative way for the observed renoprotective effects of NDUFV1. However, the underlying mechanism remains unclear.

Overall, the present study showed that increased expression of NDUFV1, a member of the mitochondrial complex I, is an effective approach against kidney dysfunction in AKI. At this regard, NDUFV1 plays a pivotal role for improving mitochondrial function and homeostasis in the kidney. By considering the kidney is an energy‐demanding organ, maintaining mitochondrial integrity, and thus providing sufficient energy are of great importance for the proper functioning of the kidney. Mitochondrial complex I is an entry of proton into electron transport chain to produce ATP, and it plays a pivotal role in energy supply. Therefore, targeting mitochondrial complex I (such as NDUFV1) should be a promising way for dealing with kidney diseases including AKI.

## AUTHOR CONTRIBUTIONS

**Lu Li:** Data curation (equal); formal analysis (equal); writing – original draft (equal). **Lingling Zhang:** Data curation (equal); formal analysis (equal); writing – original draft (equal). **Yingjie Cao:** Formal analysis (supporting); methodology (supporting). **Xu Chen:** Methodology (equal); resources (supporting). **Haifeng Gong:** Methodology (equal); writing – review and editing (supporting). **Yidan Ma:** Resources (equal); validation (equal). **Yuanyuan Gui:** Methodology (equal); writing – review and editing (supporting). **Tianya Xiang:** Writing – review and editing (supporting). **Jianxing Liu:** Writing – review and editing (supporting). **Xinzhong Huang:** Conceptualization (lead); funding acquisition (lead); supervision (lead); writing – review and editing (lead).

## CONFLICT OF INTEREST STATEMENT

The authors declare that they have no known competing financial interest or personal relationships that could have appeared to influence the work reported in this paper.

## Data Availability

The data presented in this study are available from the corresponding author upon reasonable request.
